# Hope as moral accompaniment: a framework for palliative care practice

**DOI:** 10.3389/fpsyg.2026.1753363

**Published:** 2026-05-01

**Authors:** Elena Ricci, Verónica Fernández-Espinosa, Claudia Navarini

**Affiliations:** 1 Department of Health and Life Sciences, European University of Rome, Roma, Italy; 2 Institute of Law, Politics and Development, Sant’Anna School of Advanced Studies, Pisa, Italy; 3 Centro de Educación en Virtdes y valores, Universidad Francisco de Vitoria, Pozuelo de Alarcón, Spain

**Keywords:** accompaniment, character education, HCPs practice, hope, hope therapy, palliative care, virtue ethics

## Abstract

Growing evidence underscores the importance of hope in shaping patients’ experiences. In the context of illness, hope regulates emotions, supports resilience, helps patients face uncertainty, and sustains the belief that life remains meaningful and worth living, even in adversity. While its relevance is well documented, very few approaches actively cultivate hope for its possible indirect therapeutic impact at a psycho-physical level. Among them, some positive psychology contributions seems to be particularly significant. Drawing on Snyder’s model, hope is conceptualized as the combination of agency—the motivation to pursue goals—and pathways thinking—the perceived capacity to identify and adapt routes toward these goals. Specifically, hope therapy exhibits significant effectiveness in chronic illness, which requires the ability to reorganize one’s activities and goals, although its clinical applications remain largely confined to cancer care. This paper argues for a broader application of hope therapy within palliative care, a field marked by complex, multidimensional vulnerability. Palliative care itself can be understood as a paradigmatic form of accompaniment: an ongoing, compassionate presence that supports patients and families. Within this relational framework, hope, understood as a moral virtue, becomes a central element, sustaining meaning, purpose, and dignity. The aim of this paper is thus to illustrate the role of the virtue of hope within the experience of illness and to argue that fostering hope in palliative care can enhance patients’ quality of life and psychosocial wellbeing. It also aims to examine the theoretical foundations and practical applications of Snyder’s hope-based intervention, highlighting its relevance in this context. Finally, it explores how cultivating hope through professional education can sustain compassionate accompaniment within palliative care systems. In this perspective, caring for vulnerability and creating new protocols for strengthening patients’ character—so that they may experience greater wellbeing even in the face of serious illness—can be understood as an expression of shared civic responsibility, involving healthcare professionals, institutions, and the wider community in a collective commitment to care.

## 1 Introduction

Growing evidence has shown the importance of hope in shaping patients’ experiences (Mardhiyah et al., 2020; Salimi et al., 2022; Ein Gal et al., 2025). The more hopeful the patient, the more compliant and oriented toward quality-of-life improvement (Snyder et al., 1991b). In the context of chronic or terminal illness, in particular, some approaches recommend actively cultivating hope for its possible indirect therapeutic impact at a psycho-physical level (e.g., Dufault and Martocchio, 1985; Herth, 1990). The contribution of Charles Snyder’s work may be particularly significant, since it offers a flexible conceptualization that can be further specified and applied to different settings, adapting the core features of hope. According to Snyder’s model, hope can be conceived as the combination of two elements: *agency*, which is the motivation to pursue goals, and *pathways thinking*, which is the perceived ability to identify and adapt routes toward goals (Snyder et al., 1991a). Although this model was conceived for educational settings, Snyder’s conceptualization may be particularly relevant also in contexts of illness (Gum and Snyder, 2002), including experiences of chronic-degenerative and chronic-terminal diseases, as the literature seems to increasingly confirm (Howell et al., 2015; Molaei et al., 2017; Azimian et al., 2021; Jahantigh et al., 2023; Azdanloo et al., 2025). Despite the limitations of the “sick condition,” indeed, the implementation of hope makes it feasible to partially transcend the situation and project the person beyond the sickness. This may occur in many different ways: through the establishment of new goals; through the identification of alternative ways of achieving a certain project, aspiration, or desire; and even through the preservation of meaning and purpose in one’s life until its end. By doing so, the person can become fully capable of exercising a renewed form of agency, which, although different from that possessed prior to illness, is no less valuable. This articulation can root and explain what other scholars have called the inner core of hope, as opposed to external hopes (Herth, 1989, 1990) or generalized and particularized hopes (Dufault and Martocchio, 1985).

As is well known, Snyder’s group validated scales that measure hope as a character strength -the Adult Hope Scale (AHS) and the State Hope Scale (SHS)—and a specific intervention: hope therapy (HT). This paper argues for the broader application of HT in palliative care, considering HT’s potential in cross-situational contexts and its adaptability to challenging environments. Palliative care—a field marked by complex and multidimensional vulnerabilities—can be understood as an extensive form of accompaniment, providing ongoing compassionate support to patients and families not only in terminal illness, but also in chronic disease, disability, and advanced old age. The practice of accompaniment has emerged as pivotal to caring and ethical practice, grounding clinical support in proximity and sustained presence through vulnerability and uncertainty (e.g., Guité-Verret et al., 2023; Nicholson et al., 2024). Within this relational framework, hope, especially when understood as a moral virtue, can become a central resource that sustains meaning, purpose, and dignity.

Our aim is twofold: first, to illustrate the role of hope in the context of illness, particularly in palliative care. Fostering hope in adult palliative care can significantly improve patients’ quality of life and psychosocial wellbeing, as well as foster realism and the ability to make informed decisions (Navarini, 2022; Navarini and Ricci, 2024). Second, we aim to examine the theoretical foundations and empirical evidence underlying Snyder’s hope-based model, highlighting its relevance in the context of early palliative care. Integrating cognitive-motivational approaches to hope with contemporary developments in virtue ethics, character formation, and moral education suggests that hope can bridge the gap between psychological wellbeing and moral growth. This provides a comprehensive framework for care that addresses the emotional and existential dimensions of illness.

The paper is structured as follows. Section two examines the psychological and philosophical underpinnings of hope and motivates the adoption of a virtue-based account of hope, specifically as a moral virtue. It further clarifies the nature and role of virtues. Section three analyses the role of character and hope as a moral virtue in the lived experience of illness. Section four focuses on Snyder’s account of hope and discusses strategies for cultivating it in the context of early or simultaneous palliative care, particularly with reference to HT. Section five considers the educational application of HT in clinical practice, especially in cases of chronic and terminal illness. It assesses the intervention’s benefits, challenges, and limitations while suggesting future educational programs. The Discussion section concludes that integrating a hope-based intervention, such as Snyder’s HT, into palliative care in an ethically grounded manner is necessary to address the multidimensional needs (physical, psychological, social, and existential) of individuals with chronic and degenerative diseases. Accordingly, the effective integration of HT in palliative care hinges on healthcare professionals’ capacity to embody hope as a virtue, making reflective self-work, character formation, and ethical engagement essential components of both clinical practice and professional education. Section seven builds on the conceptual and clinical analysis to propose a set of recommendations for education.

## 2 Hope as a virtue

For a long time, hope has been considered either an emotion, often connected to courage through the concept of confidence (Aristotle, 2009), or a theological virtue (Aquinas, 1981), lacking consideration in the realm of (human) virtues. Also in the contemporary scenario, hope is often approached as an emotion or a coping mechanism (Herth, 1989, 1990). However, research about human character has been reconsidering hope as a psychological and ethical construct which can be successfully included among the virtues (e.g. Snow, 2018) or the character strengths (Erikson, 1959; Peterson and Seligman, 2004), with a specific and significant relevance as (i) a dispositional stable trait and (ii) as a situation-related state (Snyder, 1994, 2000).

The philosophical and clinical literature increasingly recognizes it as a motivational and existential force that can be cultivated (Duggleby et al., 2012). In this dual function, hope help the agent confront uncertainty and maintain the conviction that life is worth living. The development and acquisition of such deep awareness rely on a cognitive-intellectual dimension, yet it is also a moral task. It is reasonable to view hope as neither a given nor a natural quality possessed by a select few. Instead, as Snyder suggests, hope can be cultivated by almost anyone, regardless of specific conditions. In this respect, hope’s ancient debt to emotions and theology is not dismissed but expanded and integrated in a deeper understanding of it through the lens of virtue. As a matter of fact, the contemporary notion of virtue—derived from Aristotle—includes the emotional dimension and the transcendent sense of purpose, explaining why nowadays hope is considered crucial among the virtues (Damon, 2009).

Admittedly, hope shares with any virtue the general features of rationality, stability, habit, and lying in a mean. It is related to future plans, desires and wishes. Emotionally, it refers to the perception of the future; theologically, it regards the expectation of eternal life; and ethically, it is connected with the rational and stable ability to look beyond the present in order to plan and pursue personal and social goals. It is important to note that the virtue of hope has a transcendent nature because it appeals to the future, displaying a “beyond-the-present” feature (Schmid and Lopez, 2011; Schmid et al., 2011), and because it requires the capability to overcome the boundaries of our self-centered world to see and appreciate the good things around us, displaying a “beyond-the-self” feature (Callina et al., 2015). In sum, hope can be clearly understood as a virtue that organizes cognition, affect, and agency toward a meaningful future, even under conditions of suffering and uncertainty (Kristjánsson, 2018; Sreenivasan, 2020).

This framework provides a coherent conceptual basis for interpreting hope in palliative care contexts, where clinicians are called not simply to elicit positive feelings, but to accompany patients in sustaining truthful, action-oriented, and relationally grounded forms of hope during periods of profound existential upheaval. Likewise, patients themselves are not merely passive recipients of care; they can actively contribute to sustaining hope by reconfiguring their goals, pathways, and rearticulating meaning.

In addition, because palliative care raises ultimate questions of meaning and finitude, it is important to note that, within Christian virtue ethics, hope is also classically understood as a theological virtue oriented toward ultimate fulfillment and grounded in trust in divine assistance—an understanding that, for religious and spiritual patients, can shape hope near the end of life without collapsing into denial of medical reality (Aquinas, 1981; Benedict, 2007).

At the same time, both positive-psychology commentary and clinical ethics have cautioned against treating hope as an unambiguously positive phenomenon. Tomasulo discusses how “false hope” and forms of toxic positivity can pull people away from realistic appraisal and impede adaptive coping, offering practical markers for distinguishing realistic from unrealistic hope (Tomasulo, 2020, 2023, 2026). In healthcare ethics, this concern is sharpened into the claim that endorsing or collaborating in false hope can generate concrete harms, including distorted decision-making, burdensome or non-beneficial interventions, and erosion of trust, especially when prognostic realities are avoided (Eijkholt, 2020). Palliative care scholars similarly distinguish therapeutic or “deep” hope from clinically problematic “false hope,” stressing that the relevant issue is often not mere improbability but the harmful effects of hope when it becomes disconnected from truthful communication and appropriate preparation for likely outcomes (Coulehan, 2011). From a virtue-ethical perspective, these critiques do not undermine hope as such; rather, they reinforce the need for virtuous hope, regulated by practical wisdom and oriented toward truth, thereby avoiding both despair and presumption/false optimism.

Now that we have clarified why it makes sense to speak of hope as a virtue, it is appropriate to briefly return to what a “virtue” is. Virtues can be understood as stable and educable dispositions of character that shapes how individuals perceive, feel, deliberate, and act in relation to what they take to be good (Aristotle, 2009; Kristjánsson, 2015; Lamb et al., 2022). In an Aristotelian register, virtues are not merely patterns of thought, habits, or behaviors, nor are they episodic emotions or moods. As such, virtues are expressed through characteristic patterns of thought, affective response, and action tendencies that become increasingly stable through practice, reflection, and habituation (Aristotle, 2009).

Contemporary moral psychology converges with this account by understanding virtues as psychologically instantiated patterns (including appraisals, emotional dispositions, motivational orientations, and behavioral regularities) rather than as isolated traits or purely internal states. From this perspective, virtues function as organizing structures that integrate emotion and reason in morally salient contexts (Kristjánsson, 2018). This integrative view shows that virtues may be both ethically normative and empirically tractable: they are evaluative dispositions oriented toward the good, yet expressed through cognitive-emotional processes that can be observed, supported, and cultivated through education and practice.

This virtue-ethical account is compatible with, but not reducible to, positive-psychological operationalizations of hope. Within the VIA framework, hope is identified as a character strength belonging to the virtue of transcendence (Peterson and Seligman, 2004), while Snyder’s theory specifies trainable cognitive-motivational components (agency and pathways) that can be understood as proximate skills supporting the cultivation of the virtue without exhausting its ethical meaning (Snyder, 2002; Lamb et al., 2022).

## 3 Hope in the experience of illness

Having clarified how hope is conceptualized in this work, we can now turn to a more specific question: namely, what role this virtue plays within the lived experience of illness. Snyder (1994) developed one of the best-known psychological theories of hope on the basis of two fundamental characteristics: the will to pursue one’s reachable goals, called agency, and the knowledge of how to reach them, called pathways. He subsequently defined hope as “a positive motivational state that is based on an interactively derived sense of successful (1) agency (goal-directed energy) and (2) pathways (planning to meet goals)” (Snyder et al., 1991b, p. 287). Thus, “hopeful thought reflects the belief that one can find pathways to desired goals and become motivated to use those pathways” (Snyder, 2002, p. 257). This approach is helpful when facing obstacles and impediments; as shown in the literature, high-hope people are more successful in finding multiple workable alternatives to achieve goals (Snyder et al., 1998).

Being fundamentally cognitive, Snyder’s theory implies that hope is not itself an emotion; instead, positive emotions flow from it: “goal-pursuit cognitions cause emotions” (Snyder et al., 2002, p. 258). Furthermore, pathways and agency thoughts activate an iterative process, which determines a reinforcement of hopeful goal-directed thinking and a feedback process composed of the particular emotions that result from perceived successful or unsuccessful goal attainment (Snyder et al., 2018).

Chronic and degenerative diseases are undoubtedly lived by patients as deeply transformative and destabilizing events. Like other forms of prolonged suffering, such conditions can be perceived as traumatic events, altering self-perception and producing profound biographical discontinuities (Cassell, 1991). These conditions tend to disrupt what individuals have long taken for granted, undermining their sense of normalcy and radically reshaping their expectations for the future and life plans (Ricci, 2025). For this reason, experiences of illness have been described in the literature as personally transformative (Carel and Kidd, 2020), as they modify what the individual previously knew or believed about themselves, altering the framework of self-understanding and, consequently, their narrative identity (Wright et al., 2020; Corns, 2022). At the same time, they are epistemically transformative in that they disclose a new dimension of reality previously inaccessible. One can only come to know what it means and what it is like to be ill through the experience of illness itself, just as one can only come to understand what it means and what it is like to become a parent through the experience of parenting (Paul, 2014).

From this perspective, transformative experiences are not just episodes of psychological change, but also events of epistemic discovery. The epistemic dimension of this transformation has led some scholars to describe illness as characterized by ineffability. As Kidd and Carel (2018, p. 222) observe:

Sometimes, one can’t find the words, but, at other times, there really are no words—none adequate to the project of cogently conveying to others, in mutually satisfying ways, the dynamics and character of one’s new, altered ‘way of being’. It may be that certain life experiences are so unique, dramatic, or traumatic, that they are accompanied by a sense of ineffability.

During these periods of existential upheaval, the development of character strengths and virtues can play a protective and structuring role. Over the past two decades, scholars from various disciplines have examined the “edifying potential” of adverse experiences, such as living with a chronic condition. For example, Michael Brady has defended the idea that suffering in general, and specific experiences of suffering such as illness, are essential—though not sufficient—for moral growth, especially insofar as they contribute to the “cultivation and expression” (Brady, 2018, p. 89) of specific virtues. These include adaptability, creativity, humility, intimacy, and compassion (i.e., virtues of vulnerability), which, according to Brady, are typically forged through experiences of adversity and vulnerability. Other scholars have argued that, while illness is frequently experienced as traumatic, it can nonetheless serve as a mediator (though not the only possible one) between vulnerability and growth in the context of the relationship between character and illness (e.g., Dekkers et al., 2005; Carel, 2019; Wright et al., 2020; Navarini, 2020; Umucu et al., 2021; Ricci, 2025). Studies in post-traumatic growth, positive psychology, and narrative medicine have also highlighted the protective role of specific virtues such as hope, resilience, patience, courage, and gratitude (e.g., Swift et al., 2002; Kim et al., 2011; Duan et al., 2015; Smedema, 2020; Zeligman et al., 2021), which can help individuals reconstruct meaning and purpose and sustain forms of wellbeing within the experience of illness.

From this perspective, suffering may be compatible with positive self-transformation (Davies, 2012; Brady, 2018; Tate and Pearlman, 2019; Tate, 2022, 2025). This is not to suggest, however, that suffering is always self-transformative, nor that experiences such as illness inherently promote flourishing. However, as shown by the literature, virtuous character can encourage adaptive coping strategies and a more positive response to illness, resulting in an enhanced quality of life (e.g., Swift et al., 2002; Kim et al., 2011; Ruini and Vescovelli, 2013). In other words, possessing certain virtues may enable patients to engage more constructively with the challenges imposed by their condition.

Virtues are also crucial to psychological resilience and wellbeing. Thus, moral character—specifically virtuous character—can shape not only one’s ethical orientation but also become a psychological and existential resource that mediates between suffering and positive adaptation. Indeed, a persevering individual is more likely to choose to leave the house and confront the dyskinesias and tremors caused by Parkinson’s disease than to withdraw into domestic isolation and develop anxiety disorders. By orienting emotions, values, and behaviors, virtues help patients reinterpret their situation, maintain positive agency, and reconstruct a coherent sense of self.

While the significance of moral flourishing is now well supported by a growing body of literature, this recognition is relatively recent. For a long time, flourishing and illness were considered mutually exclusive experiences, a notion reinforced by certain neo-Aristotelian interpretations of *eudaimonia*. The concept of moral flourishing originates from the Aristotelian tradition and refers to the objective capacity to live well. This requires one to “express at best the traits that characterize us” (Bina et al., 2024, p. 12, our translation). Speaking of “flourishing” in the context of illness may seem paradoxical. It seems counterintuitive that experiences marked by suffering, limitation, and loss could allow for positive transformation or the development of new strengths of character and virtues.

Aristotle (2009) conceived of flourishing as dependent on a set of external goods and preconditions, including health, which make virtuous activity possible. For centuries, this view has led to the implicit exclusion of illness from moral development. However, recent research in virtue ethics, moral psychology, and health humanities increasingly suggests that such transformation is possible and constitutes one of the most profound human responses to adversity (e.g., Wright et al., 2020). As Navarini writes, “if life can be good, then it must be possible for it to be so with pain, suffering, and death, from which it cannot be dissociated” (Navarini, 2020, p. 26–27, our translation).

This evidence, particularly the protective effect of virtues on psychological functioning, is relevant in palliative care, where extreme vulnerability, existential threat, and profound relational dependence are common. Palliative care is an integrated approach that seeks to improve the quality of life for patients and families facing life-threatening illnesses by preventing and relieving suffering through the early identification, assessment, and treatment of pain and other symptoms (Buckley, 2008). Within this holistic vision, alleviating physical pain and cultivating the patient’s inner world are inseparable dimensions of care.

Research indicates that patients who possess or cultivate virtues respond more adaptively to potentially traumatic experiences (Duan et al., 2015; Kidd, 2015; Wright et al., 2020); they also exhibit enhanced emotional stability, reduced anxiety, and a renewed sense of dignity (Kim et al., 2016). Furthermore, virtuous skills function as both individual characteristics and relational qualities that foster and maintain interpersonal connections with family members, caregivers, and professionals, which constitute the context of care (Ricci, 2025).

Several virtuous resources seem to play a special role in this process. For instance, trust appears to be especially important because it can foster the therapeutic relationship and enhance treatment adherence (Pellegrino and Thomasma, 1993; Navarini, 2020). This allows patients to remain open to assistance and guidance while encouraging professionals to adopt a caring attitude. Courage can also enable patients to confront pain and fear, make difficult decisions, and endure anxiety associated with bodily decline and medical uncertainty (Lebacqz, 1985; Brady, 2018; Navarini and De Monte, 2019). Gratitude is another example. It can foster recognition of valuable actions and relationships, even in tough times. This allows individuals to maintain a positive outlook on life and give meaning and dignity to their journey (Morgan et al., 2017; Kristjánsson, 2018; Gulliford, 2020). Thus, flourishing within palliative care is not an exceptional achievement reserved for the morally or psychologically strongest. Rather, it is a potential accessible to all who, with the help of others, engage in the gradual work of self-cultivation and meaning-making.

To acknowledge this possibility is not to romanticize illness: to be seriously ill is to face fear, uncertainty, and loss. Furthermore, the possibility of flourishing despite suffering seems to arise only under certain conditions. Kidd (2012) suggests that this depends on the nature of the illness—specifically, its intensity, duration, and cognitive impact—as well as the individual’s character and social context. This makes sense. First, meaningful work on one’s character seems viable when pain and suffering are controlled and not excessively intense (Fowers et al., 2017). When pain becomes overwhelming, it is difficult to imagine space remaining for reflection or moral self-work, since the individual’s focus is on finding ways to end that experience. Second, an individual’s character can play a crucial role. The presence or absence of specific traits may influence how a person responds to the challenges posed by illness. For instance, Chiara Ruini and Francesca Vescovelli found in their study on gratitude among breast cancer survivors that individuals with a sense of gratitude generally demonstrate better coping skills and are better able to find meaning in adverse circumstances (Ruini and Vescovelli, 2013). Third, the social network plays a central role. Having supportive relationships and being embedded within a caring community can drastically alter how a person responds to the challenges imposed by illness.

Though there are contexts in which this form of engagement is not possible, character development can still provide the cognitive, emotional, and moral tools necessary for living with illness and finding a sense of purpose. Within the realm of “skills for patients,” hope seems to occupy a prominent place (e.g., Lebacqz, 1985; Snyder et al., 1991a, 1991b; Campbell and Swift, 2002; Feudtner, 2005, 2009; Brady, 2018; Navarini, 2020, 2022; Navarini and Ricci, 2024; Ricci, 2024a, 2025). It is reported to be the last virtue that patients lose (Kübler-Ross et al., 1972) and one that they continue to desire and cultivate (Salamanca-Balen et al., 2021).

Obviously, cultivating hope in palliative care does not mean fostering unrealistic expectations of a cure. Rather, it entails sustaining a sense of moral agency and openness to life’s remaining possibilities. This includes helping patients recognize goals that still hold meaning for them and identifying ways to pursue those goals. Hope is not mere optimism or denial of reality; rather, it is a structured cognitive-motivational system that influences coping, behavior, and emotional regulation (Snyder, 2002; Cheavens and Whitted, 2023).

However, determining what constitutes a “realistic” or “appropriate” hope is often complex. Munday (2012) illustrates this with the example of a mother diagnosed with advanced breast cancer. Her hope to see her young children grow up might initially seem unrealistic, yet when she survives several years and this hope is realized, it can no longer be dismissed as irrational. Barilan (2012) argues that hope can remain realistic even when the probability of fulfillment is low, provided it is accompanied by awareness and acceptance. As discussed above (§2), what distinguishes virtuous hope from denial is not the likelihood of success, but rather, the lucidity with which the person continues to hope. A mother with a severe illness may hold on to the hope of seeing her child grow—and this hope can sustain her through pain and treatment—while also acknowledging her vulnerability and preparing for the possibility that her wish may not come true.

This understanding of hope aligns with Viktor Frankl’s existential perspective, which states that meaning and purpose can emerge in situations of extreme suffering (Frankl, 1985). Hope appears to be both a cognitive virtue and a spiritual orientation, which may also include an afterlife oriented dimension, enabling persons to transcend the boundaries of earthly existential ends (Scioli and Biller, 2009; Laranjeira et al., 2022).

Another important aspect that makes hope a central resource in the context of illness is its ability to regulate emotions, motivate action, and give life meaning in the face of mortality. According to Navarini (2020), this ability to manage one’s emotions and reconstruct one’s narrative identity is what makes hope a moral virtue. Indeed, as the literature has shown, virtues play a crucial role in this regard. Through these stable moral dispositions, individuals can control how they respond to reality (Mittleman, 2009), experiencing emotions for the right things, in the right way, and with the right intensity—a pattern reminiscent of Aristotle’s conception of virtue as the mean between excess and deficiency (Aristotle, 2009). This does not mean, of course, that hopeful people never experience negative emotions. There will inevitably be moments in a person’s life when events give rise to feelings such as anger or fear. However, what distinguishes the virtuous individual is the ability not to be carried away by these emotions or remain trapped in ruminative cycles, but rather to move beyond them.

Hope as a character strength has been described by positive psychology as a transcendent virtue (Peterson and Seligman, 2004) because it is “directed toward the future (beyond the present) and toward the other (beyond the self)” (Navarini and Ricci, 2024, p. 299). Through this lens, hope contributes to reestablishing coherence between one’s past, present, and anticipated future. This allows individuals to reinterpret their story within a framework that includes suffering but is not defined by it. In palliative care settings, this narrative reconstruction is essential for patients to make sense of their experience.

In sum, hope in the experience of illness represents far more than a mere coping mechanism. It is a moral-existential virtue that connects cognition, motivation, and emotion. Cultivating hope within palliative care can transform clinical encounters into spaces for not only symptom management, but also ethical and spiritual growth. In these spaces, both patients and caregivers can rediscover the possibility of flourishing in vulnerability.

## 4 Snyder’s hope therapy and its application in simultaneous palliative care

Although hope has long been recognized as a crucial factor in patients’ psychological adjustment to illness and in palliative care settings (Laranjeira et al., 2022), relatively few therapeutic frameworks explicitly aim to cultivate it, particularly in the context of palliative or end-of-life care (Salimi et al., 2022). Most psychosocial interventions tend to focus on symptom relief, emotional support, or meaning-making (e.g., Kissane, 2012; Breitbart, 2017). These are, without question, fundamental aspects of care. Yet they often address the consequences of suffering rather than the motivational and cognitive capacities that enable patients to actively reorient themselves toward life despite suffering.

In this respect, Snyder’s HT seems to offer a particularly promising and underutilized approach. Indeed, the only meta-analysis published on this topic specifically, based on 35 studies, provides evidence that dedicated interventions can effectively increase hope in palliative care patients (Salamanca-Balen et al., 2021). However, their review does not differentiate between the specific aspects of each intervention or focus on the potential of HT. Furthermore, the analysis only considers patients with cancer, even though they represent only a portion of the palliative care population.

As we have already pointed out, Snyder’s model of hope is grounded in cognitive-motivational theory, positing that human goal pursuit is sustained by two key dimensions: agency thinking and pathway thinking. Agency refers to the sense of determination and self-efficacy that energizes individuals toward their goals, while pathways refer to the perceived capacity to identify viable routes toward their attainment, even when obstacles arise (Snyder, 2002). Hope thus emerges not as a passive emotion but as an active, dynamic process involving motivation, planning, and adaptability.

According to this understanding, high-hope individuals seem to be characterized by greater psychological flexibility: they can set realistic goals, generate multiple alternative strategies, and maintain motivation despite setbacks. Conversely, low-hope individuals tend to experience goal blockages as personal failures, which can result in disengagement, despair, or emotional paralysis. Importantly, Snyder’s model integrates affective and relational dimensions, recognizing that hope is reinforced through supportive interactions and a sense of shared meaning.

HT operationalizes these theoretical premises through structured interventions designed to help individuals enhance their agency and pathway capacities. The intervention typically consists of eight sessions, each lasting approximately 2 h and delivered over 2 weeks (Gum and Snyder, 2002). The sessions guide participants through a process of clarifying goals, identifying barriers, and exploring alternative pathways, combined with reflective and narrative exercises that strengthen motivation and self-awareness. This structured process of goal clarification and pathway generation parallels the pedagogical structure of moral habituation, and through guided reflection and repeated practice during the sessions (further extended at home through assigned homework), hope is gradually internalized as a virtuous behavior.

Empirical evidence has demonstrated the potential of HT across a range of clinical and non-clinical populations, including individuals with cancer, depression, chronic illness in general, and trauma-related distress (Cheavens and Whitted, 2023). Outcomes frequently include improved psychological wellbeing, a greater sense of control, enhanced problem-solving skills, and reduced symptoms of anxiety and hopelessness. Despite these encouraging findings, however, the systematic integration of HT into palliative care remains rare.

Palliative care offers a particularly fertile ground for the application of hope-based interventions. A patient facing advanced illness often struggles with profound physical limitations and existential distress. They may feel a loss of agency, perceiving that meaningful goals are no longer attainable. As Snyder’s framework emphasizes, hope is not contingent upon the scale or external feasibility of one’s goals, but on the capacity to sustain agency and generate new pathways appropriate to one’s situation.

For example, in early or simultaneous palliative care—where supportive interventions are introduced alongside active treatment—patients may benefit from cultivating hope as a life-oriented disposition that can help reconfigure their relationship with illness. HT can help patients rearticulate what remains within reach: nurturing relationships, leaving a legacy, expressing gratitude, reconciling with others, or deepening spiritual awareness. These are not trivial or compensatory goals, but expressions of what Aristotle might call practical wisdom. In this sense, hope can be interpreted, following the Aretai Model (De Caro et al., 2024; De Caro et al., 2025).

Moreover, HT aligns with the virtue-based perspective increasingly endorsed within contemporary palliative ethics. Hope, understood as a virtue, operates as a mean between despair and unrealistic optimism. It sustains engagement with life’s possibilities without denying its limits. Within palliative contexts, this equilibrium is essential: as previously noted, the fostering of hope must not translate into the denial of medical reality, but neither should realism collapse into resignation. The therapeutic task is therefore to maintain what Barilan (2012) calls *lucid hope*—a form of hope that remains open to meaning, growth, and moral agency even within finitude.

The ethical implications of hope in palliative care extend beyond individual psychology. Hope shapes the relational and moral atmosphere of care, influencing communication, decision-making, and trust. When cultivated as a shared virtue among patients, families, and professionals, hope can transform the clinical encounter into a space of reciprocal moral growth. In this sense, HT is not only a psychological tool but also an ethical practice: it affirms the patient’s capacity for agency, dignity, and narrative authorship even when cure is no longer possible.

HT, when ethically grounded, can thus become an instrument for moral accompaniment: supporting patients in balancing truth-telling with emotional sustenance, autonomy with dependence, and realism with transcendence. This aligns with the Aristotelian notion that moral education involves learning to desire rightly, namely, to align one’s aspirations with what is good and attainable, while reserving the movement of the heart toward what transcends one’s immediate circumstances.

In other words, palliative care patients often face intertwined forms of suffering (physical decline, psychological distress, social isolation, and existential anguish), which demand integrative, person-centered interventions. Especially when palliative support is introduced early in the disease trajectory, character strengths such as hope can be cultivated to enhance overall wellbeing and reshape life narratives (Munday, 2012; Velić et al., 2023). This can represent a form of accompaniment that can foster purpose and meaning, helping patients to recognize the existential value of the time they are living and, consequently, to preserve a sense of agency.

## 5 Implementing hope therapy within palliative systems: educational applications

Based on these theoretical, clinical, and contextual considerations, the educational dimension of hope emerges as an essential avenue for sustaining and expanding the practice of hope within palliative care systems.

The question is now: can hope be taught successfully to palliative care patients? This also means identifying suitable trainers and developing training methods. So far, educational psychology and character education research have shown that hope can be taught as a cognitive-motivational skill that enhances wellbeing and perseverance in several contexts, including the psychospiritual dimension (Snyder et al., 2002; Lopez et al., 2004; Marques et al., 2011; Scioli et al., 2025). Across school and university environments, hope behaves like a teachable psychological construct: when educators explicitly coach goal setting (clear and valued goals), pathways thinking (multiple routes and contingency planning), and agency (self-efficacy and “willpower” for action), students show measurable gains in achievement, wellbeing, and persistence. For example, a 6-year longitudinal study with college students found that baseline hope predicted Grade Point Average (GPA) and graduation status, highlighting hope’s educational relevance beyond short-term mood effects (Snyder et al., 2002). Complementing this, school-based interventions—typically lasting 5–8 weeks—have demonstrated improvements in hope, life satisfaction, self-worth, and school adaptation, with some benefits sustained months later and feasible delivery by non-specialists (Marques et al., 2011).

Recent work in higher education adds qualitative depth, linking students’ lived experiences of hope to mental health and academic thriving, and recommending structured pedagogies (guided reflection on goals, iterative feedback on pathways, and scaffolding agency) as part of university wellbeing strategies (Berry et al., 2024). Hope education also appears culturally adaptable: interventions grounded in a “locus-of-hope” framework, which recognizes both internal and external sources of agency (family, peers, and community), have improved goal pursuit among adolescents in non-Western contexts, reinforcing the value of contextualized hope curricula (Embalsado, 2024). Translating this insight into palliative care training opens a new field of moral education for both patients and healthcare professionals.

According to Scioli and colleagues, it is important to design specific training for developing fundamental hope—which basically corresponds to trait hope—as a means to cope with anxiety and depression in multiple contexts. In particular, they found that both spirituality and religiosity cultivation may be associated with increased hope conceived as “prospective thinking, in interaction with the inevitable vicissitudes of life” (Scioli et al., 2025, p. 3).

If hope can—and should—be taught to patients, then practical methods for doing so must be identified, rather than merely documenting the empirical association between hope and perceived wellbeing or flourishing. Training palliative care teams, especially psychologists who work with patients’ mental suffering, offers a promising avenue. Such training would both (i) increase professionals’ awareness of hope as a psychological resource in advanced illness and (ii) provide them with concrete strategies for cultivating hope in their patients.

In this context, educating hope in palliative care necessarily involves educating professionals for self-awareness and self-development, rather than merely transmitting techniques. Because hope is relational, morally charged, and vulnerable to distortion into false optimism, professionals must be equipped to recognize their own emotional responses, expectations, and implicit biases when accompanying patients. Educational strategies aimed at cultivating hope should therefore integrate reflective practices, such as guided journaling and structured debriefing, with experiential learning, including role-playing, case-based discussions, and supervised clinical reflection (Kolb, 1984; Schön, 2017). These pedagogical approaches support the development of resilience, moral discernment, and emotional regulation, enabling clinicians to foster realistic, action-oriented hope oriented toward presence, meaning, and legacy rather than cure or denial (López González et al., 2025). In this way, hope education contributes not only to improved patient care, but also to the prevention of burnout and moral distress among palliative care professionals (Rushton, 2018).

These educational aims extend naturally into palliative care communication practices but also require dedicated training protocols that reflect the situational and relational complexity of serious illness. Clinicians must balance honesty with empathy, discern the appropriate timing for engaging patients, and adapt protocols to diverse personal and clinical circumstances. Training programs may therefore focus on helping professionals (a) elicit what patients hope for, namely, values-consistent and realistic goals; (b) co-construct diverse pathways to these goals (medical, psychosocial, spiritual); and (c) strengthen agency by identifying and reinforcing micro-efficacies, the actionable next steps available to patients and families (Back et al., 2009). These goals are highly compatible with the adaptation of Snyder’s HT to palliative care contexts, as some empirical attempts have been showing in recent years (Salamanca-Balen et al., 2021).

Importantly, such training may also enhance clinicians’ own hope. Because hope is inherently relational and prosocial—as already said, beyond the self and beyond the present (Scioli and Biller, 2009; Lopez, 2013; Navarini and Ricci, 2024)—it develops within networks of care, shared goals, and mutual trust. These elements are central to palliative care. Thus, educating professionals to foster hope in patients may naturally reinforce hopeful cognition within care teams themselves.

A structured assessment strategy can further support these educational goals. Snyder’s hope scales provide complementary insights into hope as both a relatively stable disposition and a dynamic state responsive to intervention. The AHS (Snyder et al., 1991b) measures dispositional agency and pathways, offering a baseline of professionals’ enduring hopeful orientation. The SHS (Snyder et al., 1996) captures momentary hope within specific contexts, allowing short-term changes induced by training or communication practices to be monitored. Combined, these measures enable a comprehensive evaluation of hope development among clinicians engaged in training programs.

Using Snyder’s scales in palliative care is justified on both theoretical and pedagogical grounds. HT conceptualizes hope as a set of cognitive-motivational processes that sustain purposeful action despite adversity. This makes it especially suitable for clinical education, where the aim is to help practitioners deliberately activate and model these processes. Unlike measures that treat hope primarily as an affective or existential state, Snyder’s framework operationalizes specific, teachable skills, such as identifying goals, generating pathways, and sustaining agency. Assessing outcomes with instruments aligned to this model strengthens coherence between the intervention’s goals and its evaluation, and supports the cultivation of intentional, evidence-based habits of hopeful thinking among healthcare professionals and, indirectly, their patients.

## 6 Discussion

Despite the relevance of hope in palliative care, it should be recognized that the clinical implementation of HT requires organizational and educational transformation (Ricci, 2024b). Adopting a *virtue-based model of care* implies rethinking the goals of health systems: rather than focusing exclusively on symptom management or psychological stabilization, care should aim at the *integral wellbeing* of the person, encompassing moral, emotional, social, spiritual, and relational dimensions. Practically, this requires revising care pathways and integrating dedicated approaches within palliative care that enable a sustained focus on character. Cultivating hope through HT interventions is a crucial part of this process, helping patients to foster a sense of agency and strengthen the awareness that, even in illness, life can still be shaped by goals and desires, and thus retain meaning and significance until the end.

Yet this focus on skills for patients is not sufficient. A broader cultural shift within healthcare is needed. Such a shift depends primarily on the reflective work that professionals undertake on themselves. Accordingly, what is required is not only technical training in the delivery of HT, but also a deeper recognition of the role of character and virtue (particularly hope), together with a thoughtful engagement with the ethical challenges inherent to care. Such self-work is essential not only for strengthening professionals’ capacities, but also for sustaining them amid the emotional and moral challenges that accompany palliative care practice. Palliative care providers may experience moral distress, burnout, and compassion fatigue (Rushton, 2018). Engaging consciously in the cultivation of character can protect them from emotional exhaustion and enhance their capacity to accompany patients authentically. Through reflective practice, team dialogue, and self-care strategies rooted in virtue ethics, caregivers can internalize the same values they seek to foster in their patients.

Concretely, implementing hope promotion as psychospiritual care can be operationalized through three complementary delivery formats: (i) brief HT-informed micro-interventions during routine palliative consultations (goal clarification, pathways brainstorming, identification of the next actionable step, and reinforcement of agency language); (ii) structured short cycles (e.g., 4–8 sessions) delivered individually or in small groups by a psychologist or trained clinician, coordinated with nursing and social work, and integrated with symptom management; and (iii) targeted referral to specialist spiritual care/chaplaincy when the patient’s hope is primarily shaped by questions of ultimate meaning, spiritual struggle, forgiveness, or end-of-life reconciliation. Across these formats, the intervention can be documented in the care plan (shared goals, agreed pathways, anticipated obstacles, and communication preferences), reviewed in multidisciplinary meetings, and revisited as the clinical trajectory evolves. This approach preserves the psychospiritual nature of hope while maintaining clinical structure and ethical safeguards against false hope. On this basis, we can specify how hope promotion, as a psychospiritual intervention, could be operationalized within routine palliative systems.

Integrating HT also calls for interdisciplinary collaboration between all healthcare professionals on the team, ensuring that hope-based interventions are adapted to the patient’s needs and preferences, as well as to their cultural and spiritual background. Moreover, institutional support is essential: the inclusion of hope-promoting strategies in care protocols and training curricula would represent a significant step toward a more humane and value-oriented healthcare model.

Within this perspective, simultaneous palliative care becomes a privileged context for the exercise of hope as a virtuous skill. Patients are invited to reinterpret their remaining time not as a mere prelude to death but as a morally significant space for reconciliation, gratitude, and self-transcendence. Professionals, in turn, are invited to see their work not as the management of decline but as the accompaniment of human flourishing in its most fragile form.

## 7 Recommendations for practice, education, and research

Based on the preceding conceptual and clinical analysis, we propose the following recommendations for education.

*Practice.* First, palliative services that aim to promote hope should embed hope work into routine clinical pathways (rather than treating it as an optional add-on), ensuring continuity across consultations and family meetings and aligning hope promotion with truthful communication to avoid false hope. Second, implementation should be interdisciplinary, with clear coordination among palliative physicians, nurses, psychologists, social workers, and spiritual-care professionals, so that goals, pathways, and agency are co-constructed in ways that respect patients’ clinical status, values, and spiritual background. Third, services should document hope-related goals and pathways within care plans and revisit them as illness trajectories evolve, thereby preserving agency while remaining responsive to changes in prognosis and functional decline.

*Education.* Training programs should prepare clinicians to cultivate hope as a psychospiritual and moral practice, not merely as a technique, through reflective learning and experiential components that support emotional regulation, moral discernment, and communication competence (Kolb, 1984; Schön, 2017). Second, training should include competence in identifying realistic, values-consistent goals, co-constructing multiple pathways (medical, psychosocial, relational, spiritual), and strengthening agency through actionable “micro-efficacies” (Back et al., 2009). Third, services should include simple monitoring strategies (e.g., AHS/SHS as appropriate) to evaluate changes in hope-related skills and support quality improvement in training and practice (Snyder et al., 1991b; Snyder et al., 1996).

*Research.* Future studies should (i) test adapted forms of HT in broader palliative populations beyond cancer, (ii) compare delivery formats (brief micro-interventions embedded in consultations versus structured short cycles), and (iii) evaluate outcomes at multiple levels, including patient wellbeing and meaning, communication quality and decision-making, caregiver moral resilience and burnout/compassion fatigue risks, team functioning. In addition, mixed-methods designs could clarify mechanisms (agency, pathways, relational accompaniment, and spiritual meaning) and identify moderators (diagnosis, stage, symptom burden, cultural and religious background) that shape when and for whom hope-based interventions are most effective.

Building on these theoretical and methodological foundations, the team is currently developing a structured educational intervention entitled “A Hope-Based Educational Project for Palliative Care (HOPE-PC).” This initiative aims to translate the principles of HT and character education into practical training resources for healthcare professionals working in palliative care contexts. While still in the design phase, the project represents a concrete step toward integrating the intentional cultivation of hope into palliative care education and practice, providing a framework for future empirical implementation and evaluation.

## 8 Conclusion

The findings and theoretical considerations presented suggest that hope, as both a cognitive-motivational construct and a moral virtue, can be intentionally cultivated both in patients and those who care for them. This dual cultivation—the therapeutic and the educational—extends the scope of palliative care from symptom management to moral and relational formation.

Integrating hope-based education may ensure the sustainability of hope as a “therapeutic” resource and ethical practice. Training professionals to recognize, elicit, and accompany hope prepares them to act not only as providers of care but also as educators of meaning. From a virtue-ethical standpoint, repetitive practices, feedback loops, and relational contexts of professional education mirror the Aristotelian process of habituation. In this sense, HOPE-PC is envisioned as a structured means of cultivating the “moral muscle” of hope. Such a program would aim to train caregivers not merely to understand hope cognitively but to embody it as a dispositional virtue expressed through clinical practice.

Hope formation in palliative care is inherently bidirectional. Professionals trained to guide patients in the reflective cultivation of hope simultaneously reinforce their own agency and moral resilience. Educational programs grounded in this reciprocity may therefore serve as preventive interventions against compassion fatigue and moral distress (Rushton, 2018), reinforcing both clinical efficacy and caregiver wellbeing.

Taken together, the philosophical, clinical, and educational dimensions of this work converge on a single insight: hope is best understood as a moral-educational ecosystem. It arises within relationships of accompaniment, is sustained by structured practices of reflection and communication, and is transmitted through the moral exemplarity of caregivers. Educating for hope, therefore, is not ancillary to palliative care. It is its moral core.
